# The Prognostic Significance and Potential Mechanism of Ferroptosis-Related Genes in Hepatocellular Carcinoma

**DOI:** 10.3389/fgene.2022.844624

**Published:** 2022-04-26

**Authors:** Wenli Li, Jun Liu, Dangui Zhang, Liming Gu, Hetong Zhao

**Affiliations:** ^1^ Reproductive Medicine Center, Yue Bei People’s Hospital, Shantou University Medical College, Shaoguan, China; ^2^ Medical Research Center, Yue Bei People’s Hospital, Shantou University Medical College, Shaoguan, China; ^3^ Research Center of Translational Medicine, Second Affiliated Hospital of Shantou University Medical College, Shantou, China; ^4^ Guangdong Provincial Key Laboratory of Infectious Diseases and Molecular Immunopathology, Shantou University Medical College, Shantou, China; ^5^ Department of Microbiology and Immunology, Center of Pathogen Biology and Immunology, Shantou University Medical College, Shantou, China; ^6^ Department of Traditional Chinese Medicine, Navy NO.905 Hospital, Naval Military Medical University, Shanghai, China

**Keywords:** ferroptosis, immune, hepatocellular carcinoma, prognosis, risk model

## Abstract

Ferroptosis exerts a pivotal role in the formation and dissemination processes of hepatocellular carcinoma (HCC). The heterogeneity of ferroptosis and the link between ferroptosis and immune responses have remained elusive. Based on ferroptosis-related genes (FRGs) and HCC patients from The Cancer Genome Atlas (TCGA), International Cancer Genome Consortium (ICGC), and Gene Expression Omnibus (GEO) cohorts, we comprehensively explored the heterogeneous ferroptosis subtypes. The genetic alterations, consensus clustering and survival analysis, immune infiltration, pathway enrichment analysis, integrated signature development, and nomogram building were further investigated. Kaplan–Meier plotter confirmed statistically differential probabilities of survival among the three subclusters. Immune infiltration analysis showed there were clear differences among the types of immune cell infiltration, the expression of PD-L1, and the distribution of TP53 mutations among the three clusters. Univariate Cox regression analysis, random survival forest, and multivariate Cox analysis were used to identify the prognostic integrated signature, including MED8, PIGU, PPM1G, RAN, and SNRPB. Kaplan–Meier analysis and time-dependent receiver operating characteristic (ROC) curves revealed the satisfactory predictive potential of the five-gene model. Subsequently, a nomogram was established, which combined the signature with clinical factors. The nomogram including the ferroptosis-based signature was conducted and showed some clinical net benefits. These results facilitated an understanding of ferroptosis and immune responses for HCC.

## Introduction

Hepatocellular carcinoma (HCC) is a common and highly lethal disease around the world (Kulik and El-Serag, 2019). However, high recurrence rates after resection compromise patient outcomes (Tabrizian et al., 2015). Sorafenib and lenvatinib have been approved as first-line therapy for advanced HCC patients with clinical benefits and depend on ferroptosis to fulfill their cytotoxic effects (Llovet et al., 2008; Louandre et al., 2013; Kudo et al., 2018). Sorafenib can induce ferroptosis, and ferroptosis is involved in sorafenib resistance (Li et al., 2020). Further understanding of the molecular mechanism of ferroptosis may aid in developing new therapy for sorafenib resistance (Sun et al., 2016). Immune-based therapies are emerging as promising strategies to treat HCC patients (Hilmi et al., 2019). Triggering immune response is a promising feature of ferroptosis in immune-based therapies (Gorgen et al., 2020). However, the role and potential mechanisms of ferroptosis in the immune response of HCC still remain elusive.

Ferroptosis is considered an iron-dependent, non-apoptotic type of cell death which is associated with reactive oxygen species (ROS) (Li et al., 2020). Recent research has shown that ferroptosis is involved in the pathological processes of a number of diseases (Sun et al., 2016; Capelletti et al., 2020). Many ferroptosis regulators have been reported in certain cancer cells. For example, circRNA IARS could positively regulate ferroptosis by suppressing the ALKBH5-mediated autophagy inhibition in HCC cells (Liu et al., 2020). A recent report also suggested that targeting ferroptosis could be a new promising strategy to improve sorafenib therapy (Nie et al., 2018). For instance, glutathione S-transferase zeta 1 enhanced sorafenib-induced ferroptosis in HCC cells (Wang Q. et al., 2021). Thus, further studies on the relationship between ferroptosis-related genes and HCC prognosis are needed.

In this study, we utilized publicly available gene expression datasets. Then, we revealed three distinct ferroptosis-related patterns, and these three patterns presented significant differences in immune cell infiltration and tumor mutations burden, suggesting that the ferroptosis-related genes exert a central role in the generation of the tumor immune microenvironment. Finally, we further constructed a five-gene signature to quantify the ferroptosis-related pattern in individual patients.

## Materials and Methods

### Data Acquisition

The gene expression microarray and corresponding clinical information were acquired from The Cancer Genome Atlas-Liver Hepatocellular Carcinoma (TCGA-LIHC, https://cancergenome.nih.gov/). To validate the prognostic potential of the genetic risk score, the independent HCC datasets were obtained through the International Cancer Genome Consortium (ICGC, https://icgc.org) and the Gene Expression Omnibus (GEO) database (GSE14520, Affymetrix HT Human Genome U133A Array, GPL3921). The protein expression profiles of HCC were acquired from The National Cancer Institute’s Clinical Proteomic Tumor Analysis Consortium (CPTAC, https://cptac-data-portal.georgetown.edu/). The ferroptosis-related genes (FRGs) were acquired from the FerrDb website (http://www.zhounan.org/ferrdb). All statistical analyses were performed in R version 4.0.5.

### Identification of Differentially Expressed Genes

Differentially expressed genes (DEGs) were determined using R package “limma” (Ritchie et al., 2015) based on the comparison of gene expression levels between HCC and liver tissues in TCGA cohort. The selection criteria for identification of DEGs were as follows: |logFC|>1 and adjusted *p* < 0.01. This study fully complies with TCGA publication requirements (http://cancergenome.nih.gov/publications/publicationguidelines). Kaplan–Meier survival analysis and the Cox proportional hazards model were used to analyze the association between the different clusters and prognosis with the R package “Survminer.” The receiver operating characteristic (ROC) curve was used to assess the prognosis classification performance of the signature and nomogram, and the area under the curve (AUC) was calculated using R package “timeROC.”

### Functional Enrichment Analysis

The R package “clusterProfiler” was used for Gene Ontology (GO) and KEGG pathway analysis (Yu et al., 2012). *p* < 0.01 and FDR <0.05 were considered statistically significant. Gene set enrichment analysis (GSEA) of hallmark gene sets was acquired from MSigDB database v7.1 (Hänzelmann et al., 2013; Subramanian et al., 2005).

### Mutation Analysis

The genomic mutation data were curated from TCGA database. The copy number variation distribution information of FRGs on different chromosomes was plotted using the R package “Rcircos.”

### Subclusters and PCA Analysis

To study the function of DEGs, we separated patients into three subclusters by the R package of “ConsensusClusterPlus” (Wilkerson and Hayes, 2010). The R package “ggplot2” of principal component analysis (PCA) was carried out to explore the genes in subclusters of HCC.

### Immune Cell Infiltrated in Different HCC Clusters

The enrichment scores of immune infiltration levels were calculated by single sample gene set enrichment analysis (ssGSEA). ssGSEA is a popular enrichment algorithm, which was extensively utilized in medical studies (Liu et al., 2021a; Liu et al., 2021b; Liu et al., 2022a; Liu et al., 2022b; Liu et al., 2022c). Data were visualized using multidimensional scaling (MDS) and a Gaussian fitting model. The immune infiltration assessment was performed using the Tumor IMmune Estimation Resource (TIMER; https://cistrome.shinyapps.io/timer/) and microenvironment cell population count (MCP-counter) method.

### Building a Combined Nomogram for Clinical Practice

In the present study, a nomogram was constructed based on the risk model and pathological stage to assess the probability of individual patient’s OS with HCC. Meanwhile, the calibration curves were performed to verify the accuracy of the nomogram. Furthermore, the ROC and DCA were applied to evaluate the prediction efficiency and net benefit of the nomogram for HCC patients at 1, 3, and 5 years.

## Results

### Identification of Ferroptosis-Related DEGs in Patients With HCC

**FIGURE 1:**
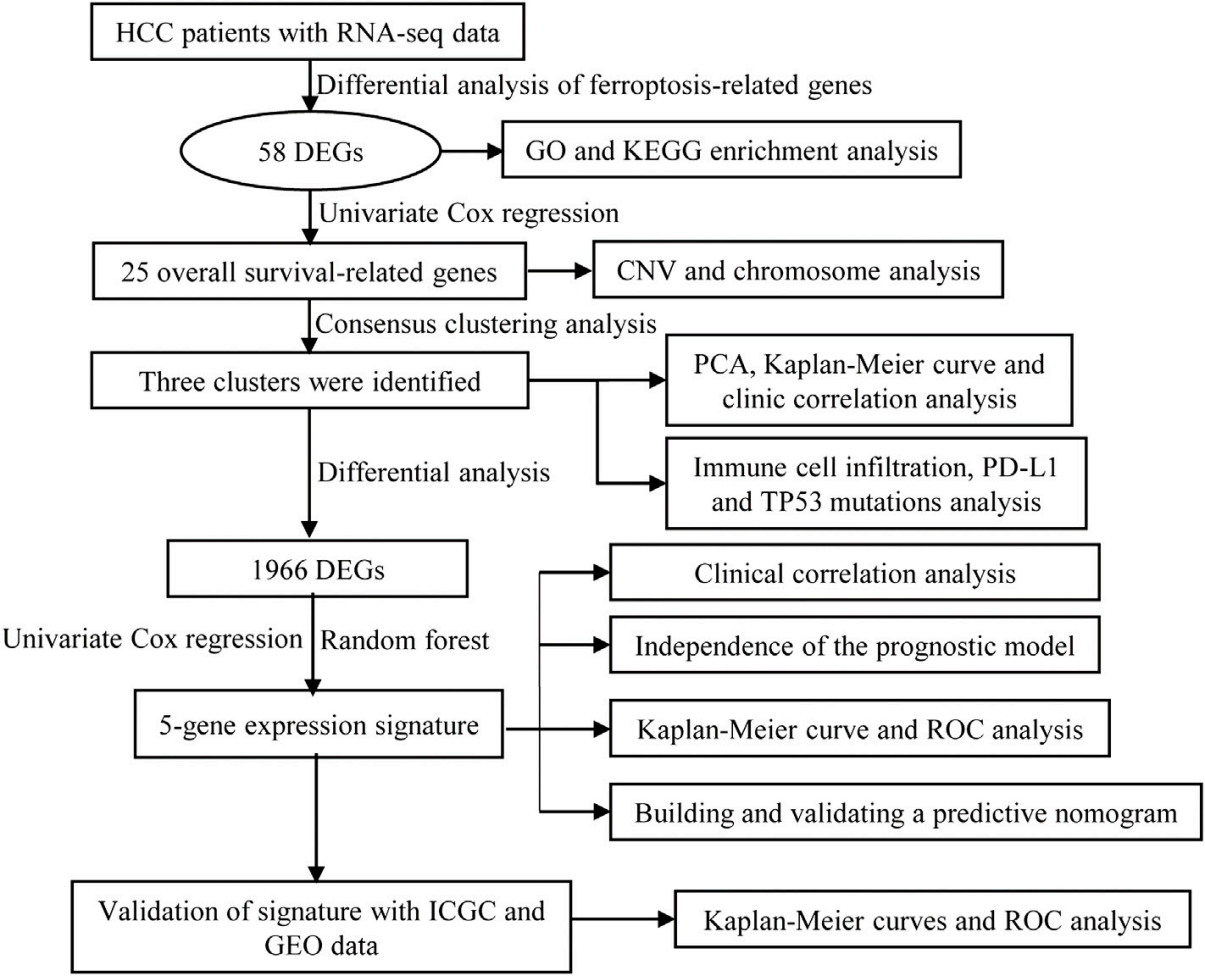
Flow diagram of the study.

**FIGURE 2:**
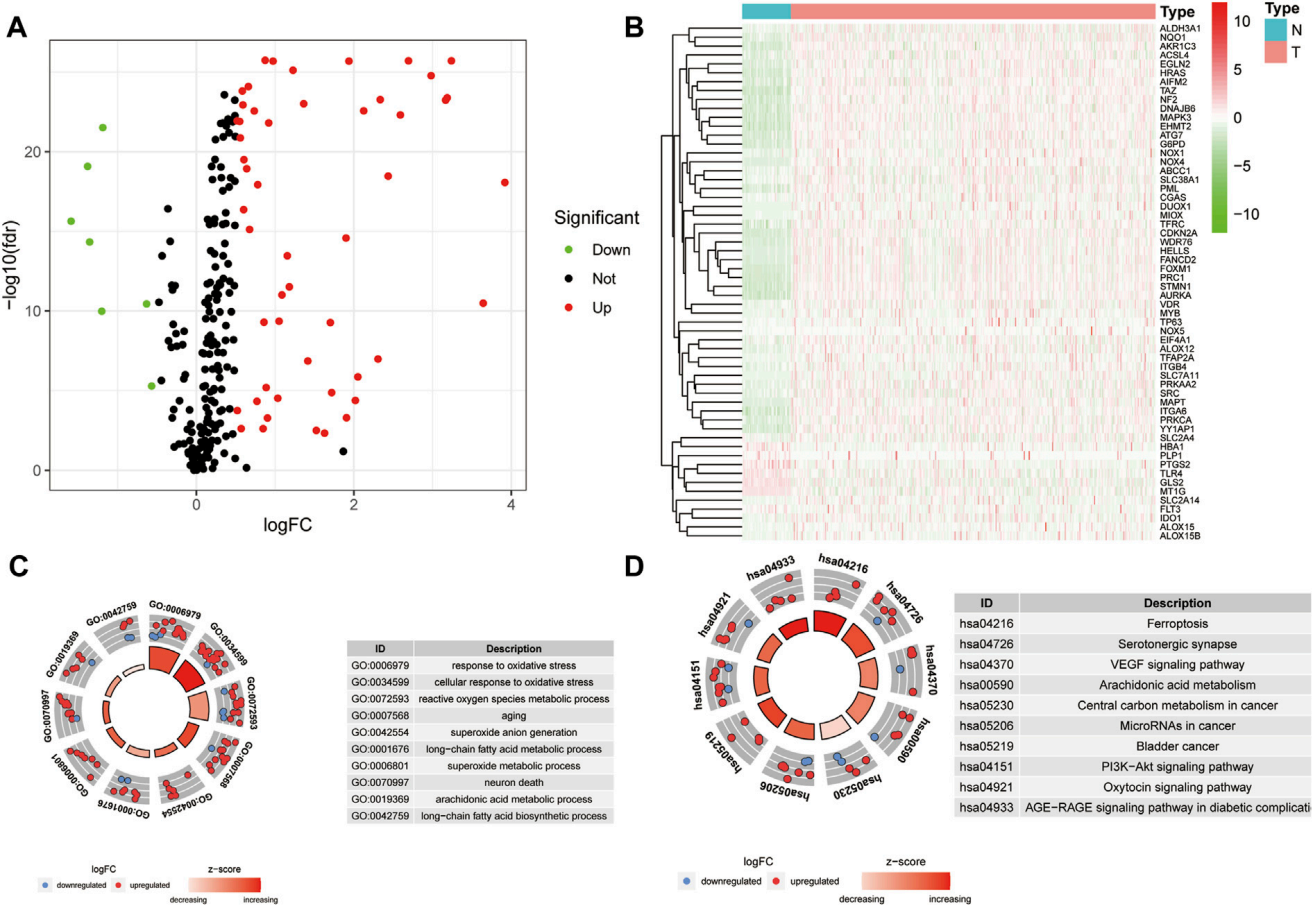
Differential analysis of ferroptosis-related genes. **(A)** Volcano map of ferroptosis-related DEGs for TCGA samples (FC > 1.5, FDR < 0.01). **(B)** Heatmap of ferroptosis-related DEGs. **(C–D)** GO **(C)** and KEGG **(D)** analysis of ferroptosis-related DEGs.

A workflow of this study is presented in Figure 1. Based on the threshold of FDR <0.05 and |log2 FC| > 1, a total of 51 upregulated and 7 downregulated significant ferroptosis-related differentially expressed genes (DEGs) were identified (Figure 2A), and the expression landscape of DEGs was shown in the heatmap (Figure 2B). Next, the ferroptosis-related DEGs were selected to perform the GO (Figure 2C) and KEGG (Figure 2D) analyses. Subsequently, several biological processes, including response to oxidative stress, reactive oxygen species metabolic process, and aging, were detected in GO terms. As to KEGG pathway analysis, ferroptosis, serotonergic synapse, VEGF signaling pathway, arachidonic acid metabolism, etc. were mostly associated with these genes.

### Landscape of Genetic Alterations of Ferroptosis-Related DEGs in HCC

**FIGURE 3:**
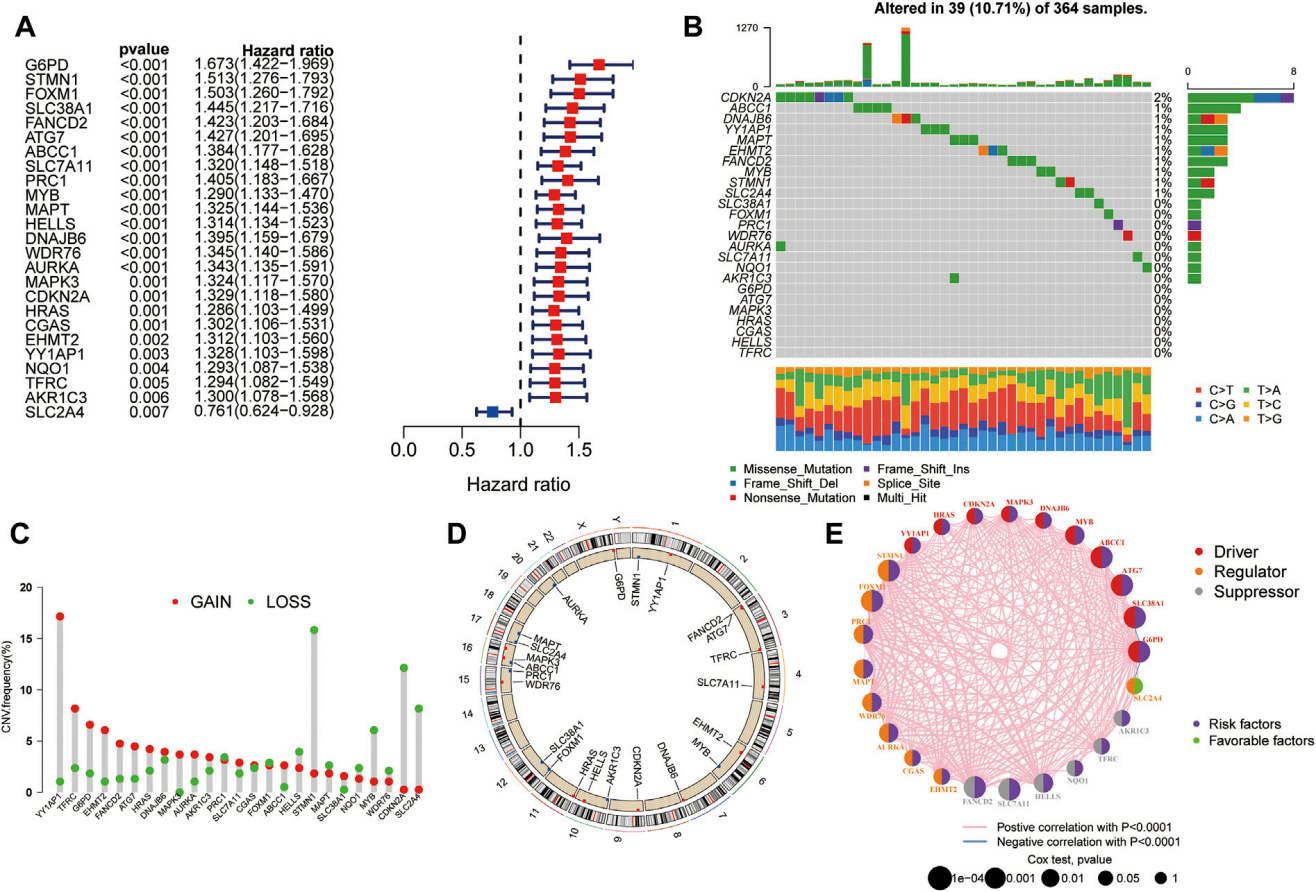
Landscape of genetic alterations of ferroptosis-related DEGs in HCC. **(A)** Identification of genes associated with overall survival via the univariate Cox regression analysis. **(B)** A total of 39 of the 364 HCC patients experienced genetic alterations of 25 ferroptosis-related genes, with a frequency of 10.71%. **(C)** The landscape of mutation frequency of 25 ferroptosis-related genes in HCC patients. The column represents the alteration frequency, the green dot represents the deletion frequency, and the red dot represents the amplification frequency. **(D)** Location of CNV alteration of ferroptosis-related genes on chromosomes. **(E)** PPI network of ferroptosis-related genes.

Univariate Cox regression analysis was performed to evaluate the prognostic value of ferroptosis-related DEGs, and 25 DEG aberrant expressions were correlated with the overall survival of HCC patients in TCGA cohort (Figure 3A). Mutation analysis of 25 prognostic genes revealed that 10% of genes were mutated by cBioportal (Figure 3B). We first figured out the somatic mutation rates of 25 DEGs based on the simple nucleotide variation data from TCGA database. The results revealed that only 39 of the 364 (10.71%) samples presented genetic alterations. We observed CNV changed in YY1AP1, STMN1, CDKN2A, and SLC2A4 (Figure 3C). The location of 25 ferroptosis-related genes on the chromosomes is shown in Figure 3D. The PPI network of ferroptosis-related DEGs is constructed in Figure 3E.

### Consensus Cluster and Survival Analysis

**FIGURE 4:**
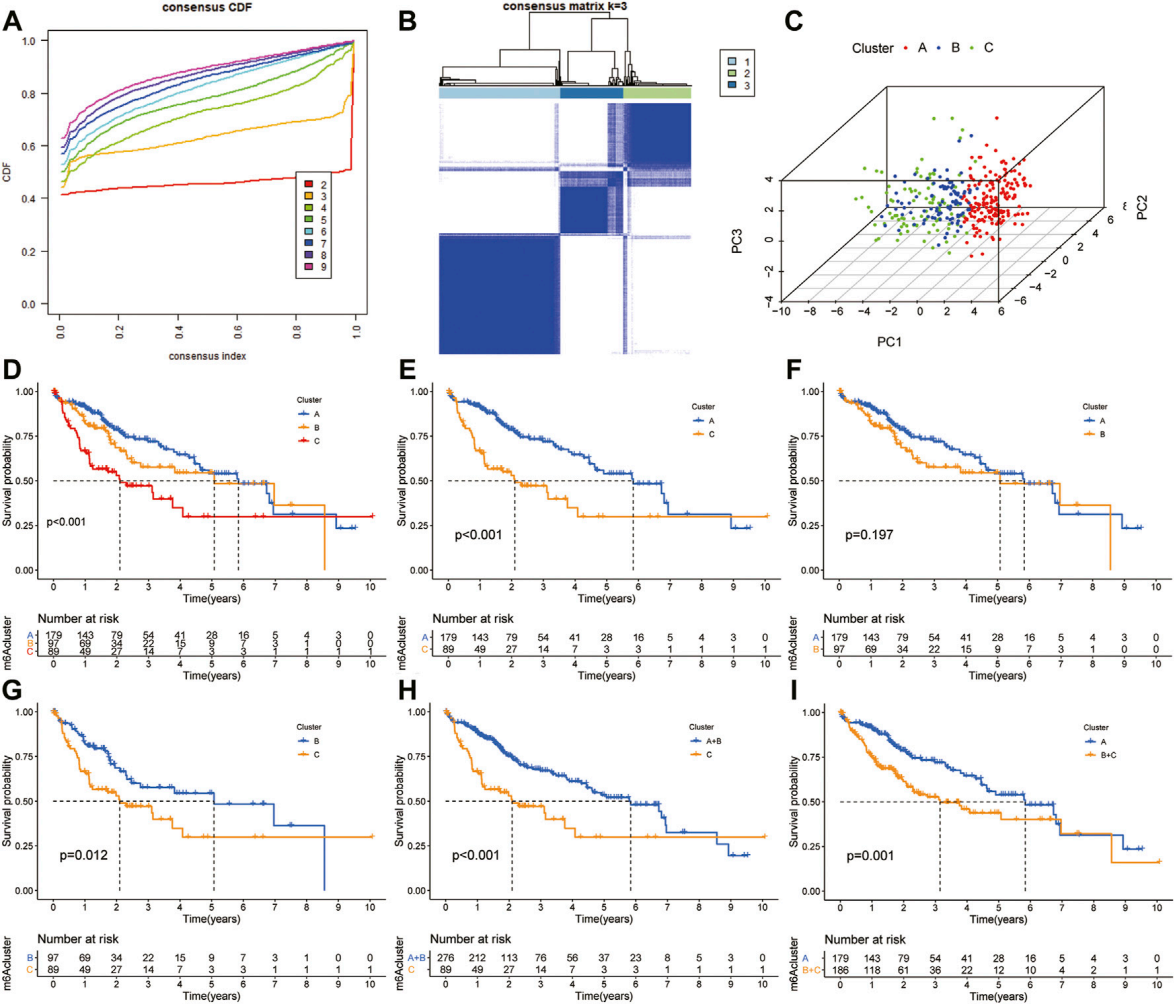
Subclusters and survival analysis based on the expression level of DEGs. **(A)** Classification of consensus clusters. The cumulative distribution function (CDF) was set from k = 2 to 9. **(B)** Consensus matrix of three subclusters (k = 3). **(C)** Three-dimensional PCA of three subclusters according to HCC patients. **(D)** Kaplan–Meier (K–M) analysis of overall survival (OS) for HCC patients in TCGA cohort with different HCC clusters. **(E)** K–M curves of OS for HCC patients in TCGA cohort with A and C clusters. **(F)** Kaplan–Meier curves of OS for HCC patients in TCGA cohort with A and B clusters. **(G)** K–M curves of OS for HCC patients in TCGA cohort with B and C clusters. **(H)** K–M curves of OS for HCC patients in TCGA cohort with A + B and C clusters. **(I)** KM curves of OS for HCC patients in TCGA cohort with A and B + C clusters.

**FIGURE 5:**
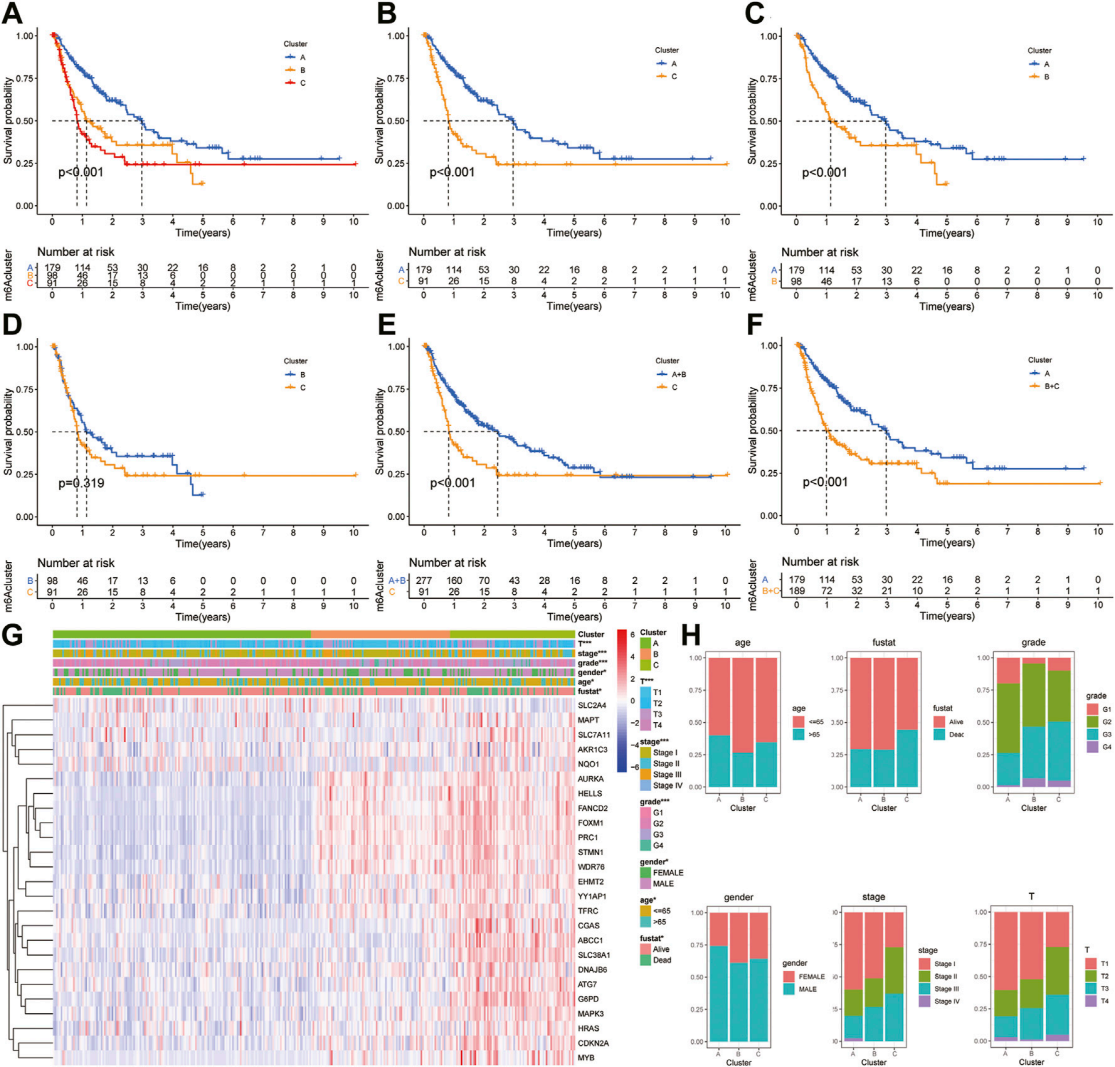
Survival analysis and clinicopathologic factors in distinct HCC clusters in TCGA cohort. **(A)** Kaplan–Meier (K–M) analysis was performed to assess the relationship between the disease-free survival (DFS) and HCC clusters. **(B)** K–M plotter of DFS for HCC patients with A and C clusters in TCGA cohort. **(C)** K–M curves of DFS for HCC patients with A and B clusters in TCGA cohort. **(D)** K–M curves of DFS for HCC patients with B and C clusters in TCGA cohort. **(E)** K–M curves of DFS for HCC patients with A + B and C clusters in TCGA cohort. **(F)** K–M curves of DFS for HCC patients with A and B + C clusters in TCGA cohort. **(G)** Heatmaps showing the relationship between clinicopathologic factors (including clusters A, B, and C) and the expression of 25 DEGs of patients in TCGA cohort. **(H)** Proportion of clinicopathologic factors (including age, gender, status, and stage) in the three clusters.

The “ConcensusCluster” package in R language was used to perform consensus clustering analyses of HCC samples from TCGA dataset based on the 25 ferroptosis-related genes shown in Figures 4A,B. In order to obtain a reliable and stability cluster, k = 3 was chosen from k = 2 to 9 in TCGA dataset. However, the PCA analysis revealed that k = 3 was also preferable (Figure 4C). Then, the HCC patients were split into three distinct clusters: A, B, and C clusters. Figure 4D shows an obvious distinction in overall survival among these three clusters. K–M analysis suggested that significant overall survival benefit of the A cluster over B or C cluster, and B cluster over C cluster (Figures 4D–G). After combining clusters A and B, a significantly longer overall survival was shown for A + B than for C by log-rank tests (*p* < 0.001; Figure 4H). In addition, after combining clusters B and C, a significantly longer overall survival was shown for A than for B + C by log-rank tests (*p* < 0.001; Figure 4I). A clear disease-free survival (DFS) difference among the three clusters was observed (*p* < 0.001; Figure 5A). The DFS was longer for cluster A than for cluster B patients, and cluster B patients than for cluster C patients. The DFS in cluster A was significantly longer than that in clusters B and C (*p* < 0.001, respectively; Figure 5B,C). The DFS was longer for cluster B than for cluster C patients (*p* < 0.001; Figure 5D). After combining clusters A and B, a significantly longer overall survival was shown for A + B than for C by log-rank tests (*p* < 0.001; Figure 5E). In addition, after combining clusters B and C, a significantly longer overall survival was shown for A than for B + C by log-rank tests (*p* < 0.001; Figure 5F). Figures 5G,H indicate that the three clusters had different clinicopathologic features, including T stage, tumor stage, grade, gender, age, and survival status.

**FIGURE 6:**
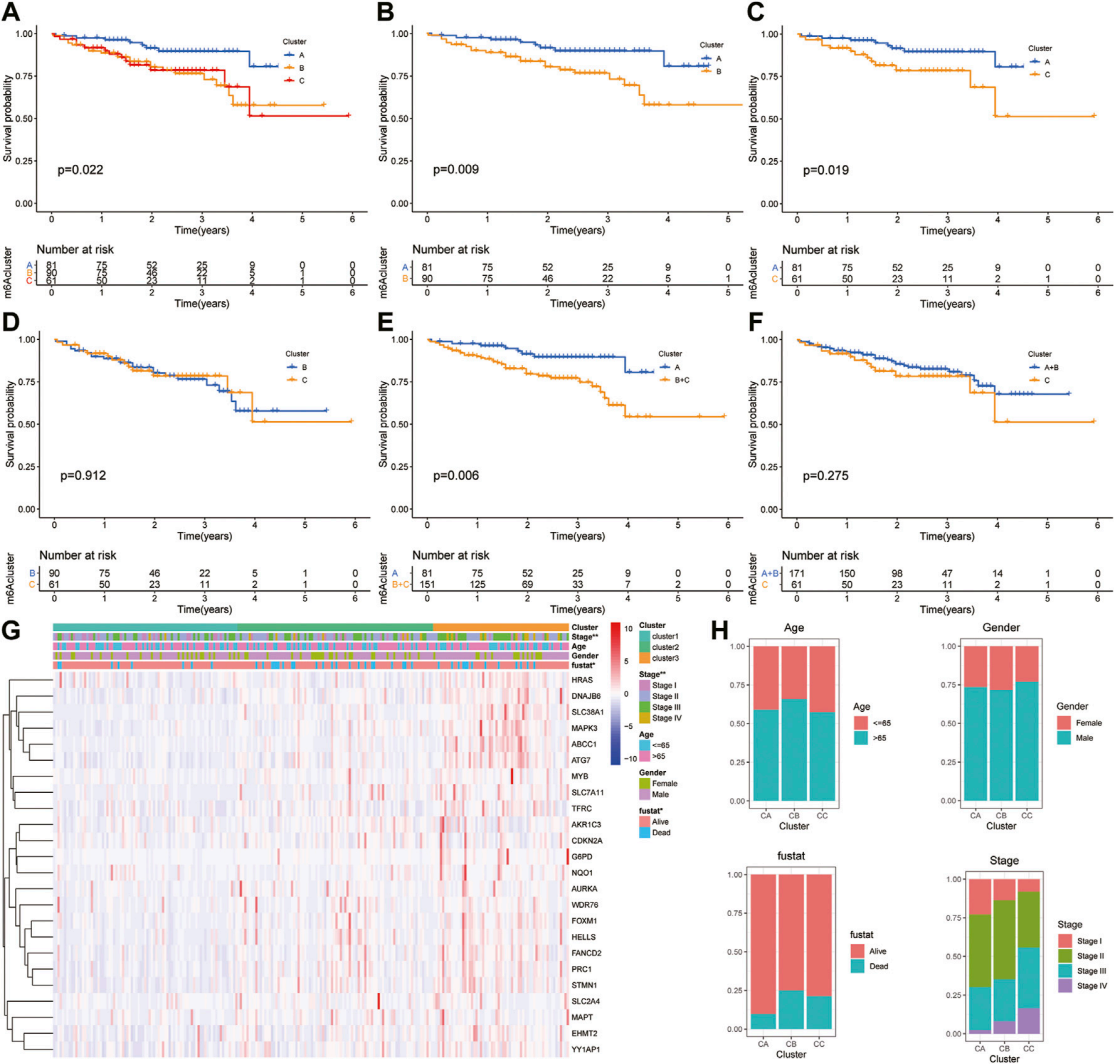
Survival analysis and clinicopathologic factors in distinct HCC clusters in the ICGC cohort. **(A)** Kaplan–Meier (K-M) analysis of overall survival (OS) for HCC patients with different HCC clusters in the ICGC cohort. **(B)** K–M curves of OS for HCC patients in the ICGC cohort with A and C clusters. **(C)** K–M curves of OS for HCC patients with A and B clusters in the ICGC cohort. **(D)** K–M curves of OS for HCC patients with B and C clusters in the ICGC cohort. **(E)** K–M curves of OS for HCC patients with A + B and C clusters in the ICGC cohort. **(F)** K–M curves of OS for HCC patients with A and B + C clusters in the ICGC cohort. **(G)** Heatmaps presenting the relationship between clinicopathologic factors (including the cluster A, B, and C) and the expression of 25 DEGs of patients in the ICGC cohort. **(H)** The proportion of clinicopathologic factors (including age, gender, status, and stage) in the three clusters.

Moreover, the ICGC cohort was regarded as the external validation set. Figure 6A shows an obvious distinction in overall survival among these three clusters (*p* = 0.022). Figures 6B,C show patients in cluster A had longer OS time than patients in cluster B (*p* = 0.009) and cluster C (*p* = 0.019). But the survival difference between clusters B and C was not significant (*p* = 0.912) (Figure 6D). After combining clusters B and C, patients in cluster A presented longer OS time than patients in clusters B + C (Figure 6E; *p* = 0.006). But the survival difference between clusters A + B and C was not significant (Figure 6F; *p* = 0.275). The analysis further showed there were significant differences in the TNM stage and survival status (Figures 6G,H).

### Immunoinflammatory Cells Infiltrating in Different Ferroptosis-Related Clusters

**FIGURE 7:**
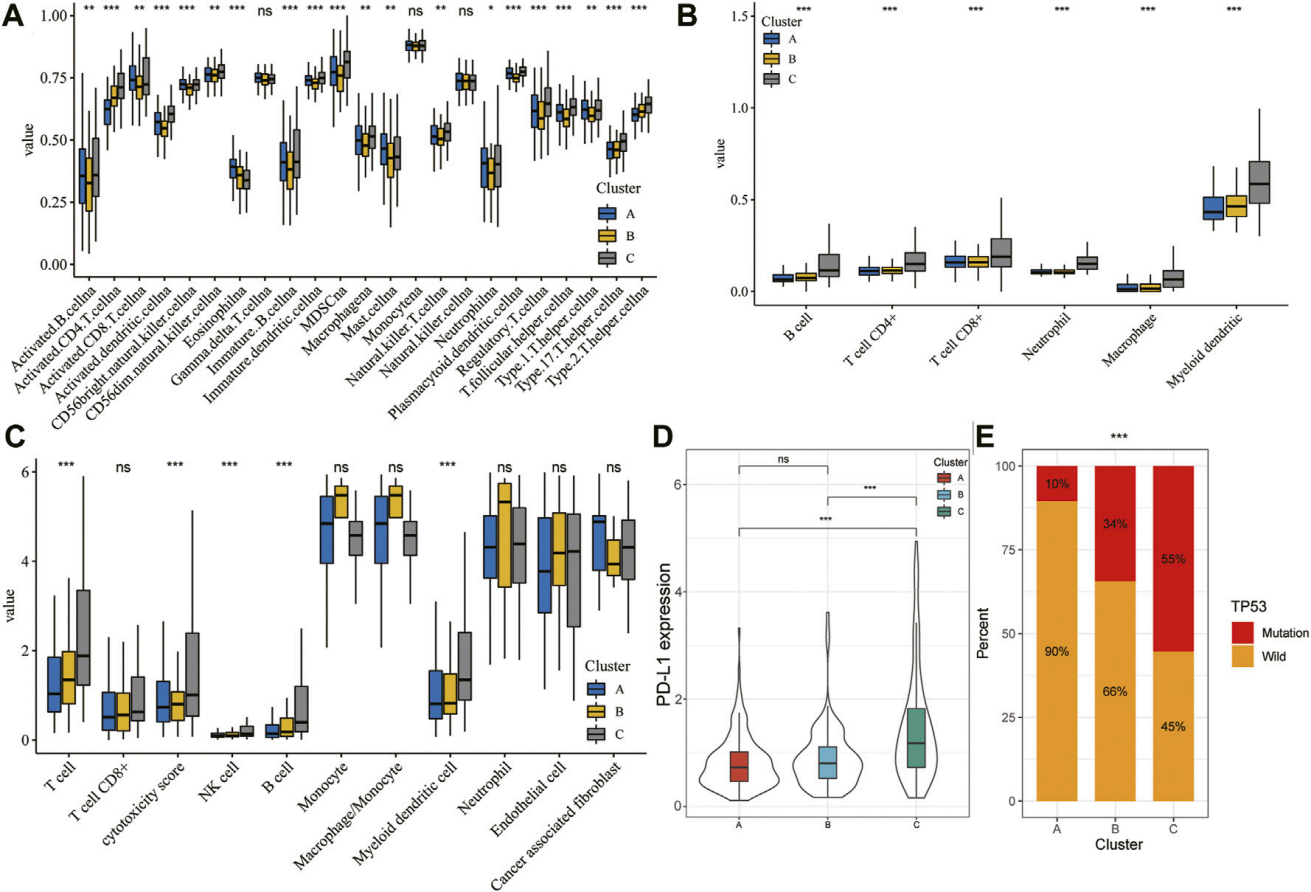
TME characteristics in different HCC subtypes. **(A–C)** Landscape of immune cells infiltration among three clusters using the ssGSEA algorithm **(A)**, TIMER algorithm **(B)**, and MCPcount **(C)**. **(D)** Violin plot showing the expression of PD-L1 in the different HCC clusters. The thick line represents the median value. **(E)** Proportion of mutation or wild TP53 in the three clusters. The statistical analysis was performed by Kruskal–Wallis H test. *p < 0.05; **p < 0.01; ***p < 0.001.

Immune cells are well known to be the key part for antitumor immune response and immunotherapy; therefore, we examined the distribution of immune cell infiltration in the three clusters based on the ssGSEA algorithm (Figure 7A). A higher ssGSEA score indicated more infiltrating immune cells. The results showed that activated CD4+/CD8+ T cells, effector memory CD4+/CD8+ T cells, activated dendritic cells, activated B cells, immature B cells, immature dendritic cells, MDSC, regulatory T cells, T follicular helper cells, and type 17 T helper cells were mainly enriched in the C subtype. However, eosinophils were markedly enriched in the A subtype. Furthermore, we also assessed the immune infiltration level using TIMER and MCPcount algorithms, which indicated a significant difference between the three clusters (Figures 7B,C; *p* < 0.05). In addition, there was clear difference in the expression of PD-L1 and TP53 between the three clusters (Figures 7D,E; *p* < 0.05).

### Pathway Enrichment Analysis of Different Ferroptosis-Related Clusters

**FIGURE 8:**
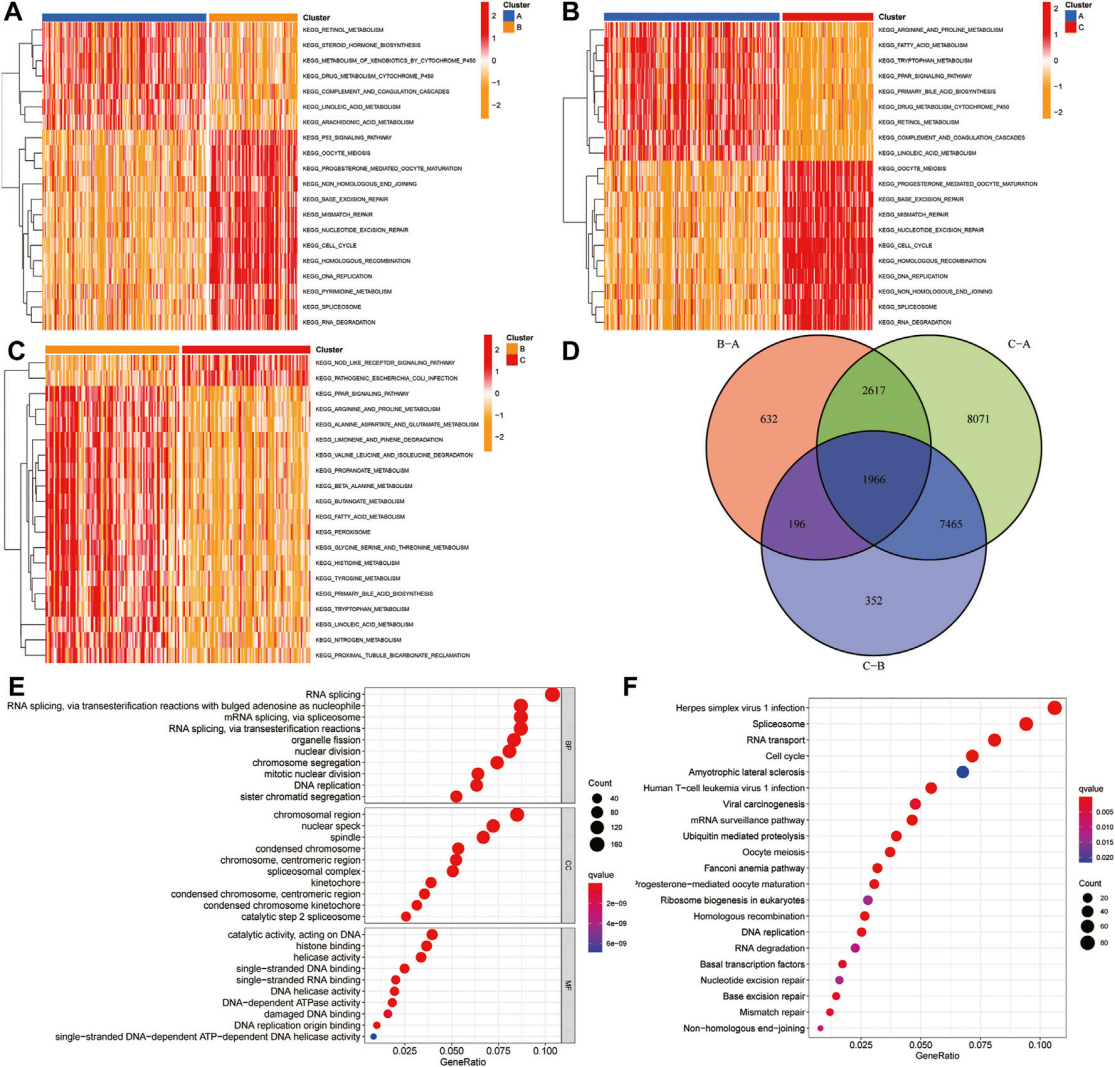
GSVA and functional analysis. **(A–C)** Heatmap of the differentially enrichment pathways between A and B clusters **(A)**, A and C clusters **(B)**, and B and C clusters **(C)**. **(D)** Venn diagram showed the common differentially expressed genes (DEGs) between three HCC clusters. **(E–F)** GO **(E)** and KEGG **(F)** analysis of ferroptosis-related DEGs. GSVA: gene set variation analysis.

To explore the biological molecular changes among these ferroptosis-related clusters, we performed GSVA enrichment. Heatmap showed that P53 pathway, DNA replication, and cell cycle were more enriched in cluster B than in cluster A (Figure 8A); cell cycle, DNA replication, and mismatch repair were more enriched in cluster C than in cluster A (Figure 8B); the and PPAR pathway, fatty acid metabolism, and multiple metabolism pathway were more enriched in cluster C than in cluster B (Figure 8C). The color changes from yellow to red, determining an increase in the value of the enriched score. Blue represents cluster A, yellow represents cluster B, and red represents cluster C. GSVA showed that multiple metabolic and cell cycle pathways were identified. A total of 1,966 differential genes were obtained among the three clusters (Figure 8D). Functional enrichment analysis was performed through the GO and KEGG (Figures 8E,F).

### Development of an Integrated Signature

**FIGURE 9:**
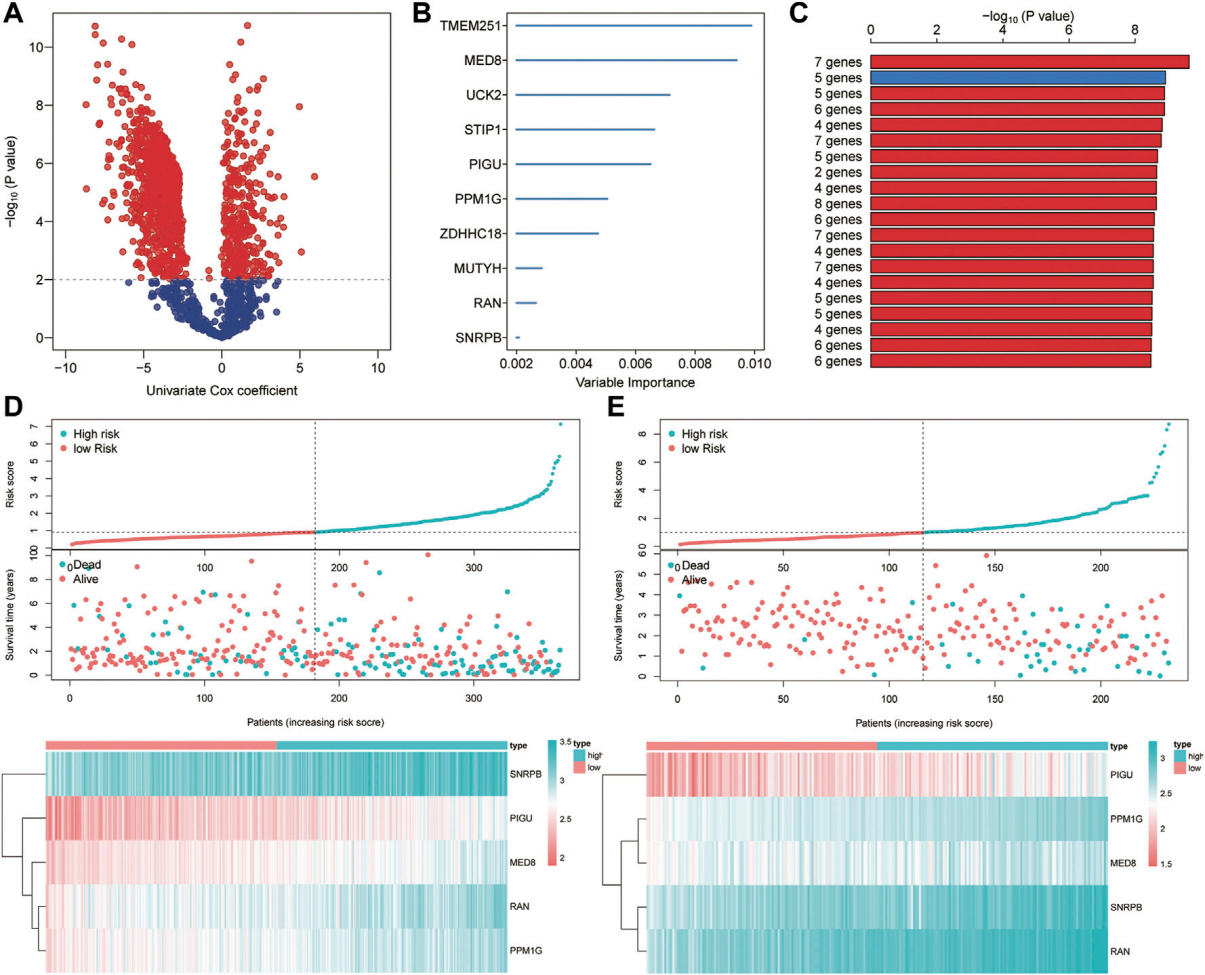
Construction of the ferroptosis-related signature. **(A)** Identification of overall survival-related DEGs via the univariate Cox analysis. **(B)** Random survival forest analysis screened 10 genes. **(C)** Screening the best risk model based on the *p* value of Kaplan–Meier, and the top 10 models were identified. **(D–E)** Distribution of the risk scores, survival status, and gene expression between high- and low-risk groups in TCGA **(D)** and ICGC **(E)** sets.

Based on the univariate Cox regression analysis of TCGA cohort, genes with significant prognostic value were further extracted (*p* < 0.01; Figure 9A). By the random survival forest variable hunting (RSFVH) algorithm, the top 10 genes were ranked based on their predictive importance: TMEM251, MED8, UCK2, STIP1, PIGU, PPM1G, ZDHHC18, MUTYH, RAN, and SNRPB (Figure 9B). We performed Kaplan–Meier analysis to figure out the best risk model with relatively small genes and relative significant *p* value. Then, we found the five-gene signature ranked top, including MED8, PIGU, PPM1G, RAN, and SNRPB (Figure 9C). Subsequently, we inspected the distribution of risk score and survival status in high- and low-risk groups, and the results demonstrated that the high-risk group presented a higher risk score and rates of death than the low-risk group (Figure 9D). The heatmap showed that the five gene had a higher expression level in the high-risk group than the low-risk group (Figure 9D). Similar results were found in the ICGC cohort (Figure 9E).

### Kaplan–Meier and Time-dependent ROC Curves of Five-Gene Signature

**FIGURE 10:**
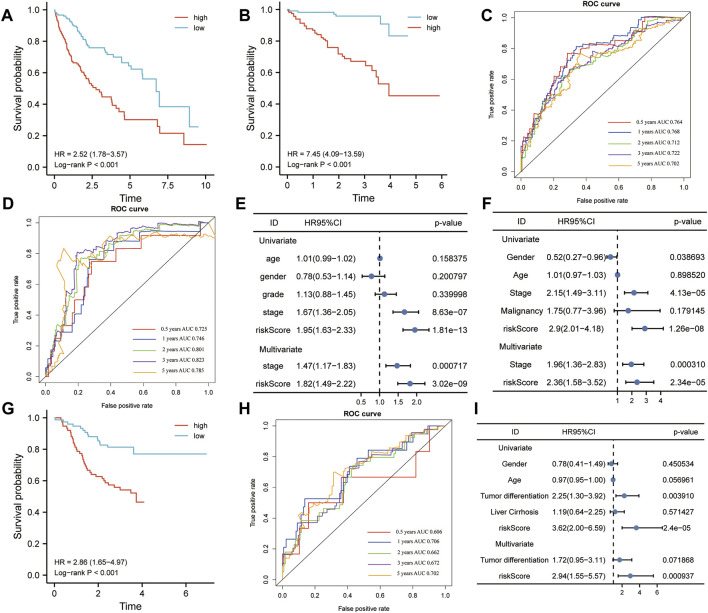
Kaplan–Meier (K-M) and receiver operating characteristics (ROC) analysis. **(A,B)** K–M plotter was applied to assess the difference in overall survival between high- and low-risk groups in TCGA **(A)** and ICGC **(B)** cohorts. **(C,D)** Area under the curve (AUC) showing the prediction power of the risk model for overall survival in TCGA **(C)** and ICGC **(D)** cohorts. **(E,F)** Univariate and multivariate Cox analyses were used to determine the prognostic value of risk model in TCGA **(E)** and ICGC **(F)** cohorts. **(G)** Difference between high- and low-risk groups in overall survival (OS) based on the protein expression levels of this signature. **(H)** AUC reporting the predictive power of the risk model in the CPTAC cohort. **(I)** Univariate and multivariate Cox analyses confirmed that the risk score was an independent prognostic factor in the CPTAC set.

The HCC patients were split into high-risk and low-risk groups by the median risk score. In the training set, K–M analysis indicated that the patients with high-risk scores had worse overall survival than patients with low-risk scores (HR = 2.52, 95% CI:1.78-3.57, log-rank test *p* < 0.001) (Figure 10A). In the ICGC dataset, the overall survival of patients with low-risk scores was significantly longer than that of patients with high-risk scores (HR = 7.45, 95% CI: 4.09-13.59, log-rank test *p* < 0.001) (Figure 10B). To further understand the predictive ability at 0.5, 1, 2, 3, and 5 years of the signature, we used time–ROC analysis in TCGA (Figure 10C) and ICGC (Figure 10D) cohorts. The AUC of the signature in TCGA cohort was 0.764, 0.768, 0.712, 0.722, and 0.702 at 0.5, 1, 2, 3, and 5 years, respectively (Figure 9C). The AUC in the ICGC cohort was 0.725, 0.746, 0.801, and 0.823, 0.785 at 0.5, 1, 2, 3, and 5 years (Figure 10D). These suggest that the risk model provided a reliable predictive efficiency in HCC prognosis. Univariate Cox analysis was performed on all factors, and multivariate Cox analysis was performed with variates which had *p* < 0.05 of the univariate Cox analyses in TCGA (Figure 10E) and ICGC (Figure 10F) cohorts. The results confirmed that the clinical stage and risk score were stronger independent poor prognostic factors in both cohorts. To further determine the robustness of the risk mode, we performed an additional analysis to predict the overall survival of HCC patients in an independent GEO cohort (GSE14520). Similarly, K–M curves showed that the patient with high-risk had worse overall survival (Supplementary Figure S1A). The AUC of the signature was 0.73, 0.671, 0.610, 0.581, and 0.606 at 0.5, 1, 2, 3, and 5 years, respectively (Supplementary Figure S1B). The univariate and multivariate Cox regression analyses demonstrated that the risk score was an independent risk factor for the overall survival of HCC (Supplementary Figure S1C). This result was validated by another independent cohort CPTAC in the protein level, which completely echoed the result that high-risk patients appeared to present a poorer overall survival than those with low-risk scores. The AUC of the signature in the CPTAC cohort is 0.606 at 0.5 year, 0.706 at 1 year, 0.662 at 2 years, 0.672 at 3 years, and 0.702 at 5 years. Univariate and multivariate Cox analyses also demonstrated that protein risk score was an independent prognostic indicator in the protein level.

### The Relationship Between the Risk Score and Clinical Pathological

**FIGURE 11:**
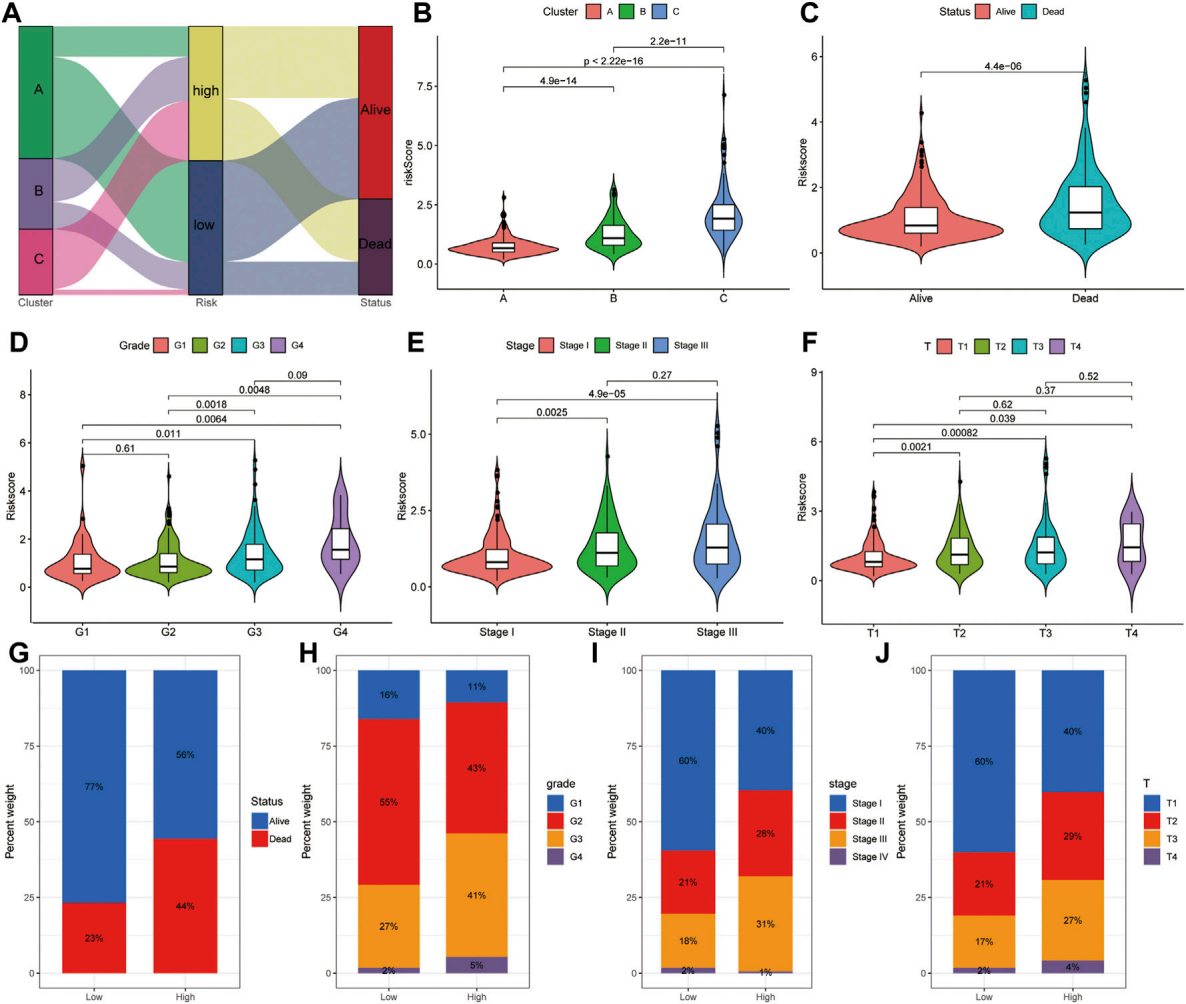
Correlation analysis between risk score and clinical features. **(A)** Distribution of different HCC clusters (A, B, and C) and risk group. **(B)** Violin plot showing risk score in different HCC subtypes. **(C)** Violin plot showing risk score in groups with alive or dead status. **(D)** Violin plot showing risk score in groups with difference grades. **(E)** Violin plot showing risk score in groups with differences in tumor stages. **(F)** Violin plot showing risk score in groups with difference in TNM stages. Violin plot showing risk score in groups with alive or dead status. **(G)** Fraction of patients with alive or dead status in low- or high-risk score groups. Alive vs dead: 77 vs 23% in the low-risk score group and 56 vs 44% in the high-risk score group. **(H)** Fraction of patients with difference grades in low- or high-risk score groups. G1 vs G2 vs G3 vs G4: 16 vs 55% vs 27 vs 2% in the low-risk score group, 11 vs 43% vs 41 vs 5% in the high-risk score group. **(I)** Fraction of patients with difference in tumor stages in low- or high-risk score groups. Stage I vs stage II vs stage III vs stage IV: 60 vs 21% vs 18 vs 2% in the low-risk score group, and 40 vs 28% vs 31 vs 1% in the high-risk score group. **(J)** Fraction of patients with difference in TNM stages in low- or high-risk score groups. T1 vs T2 vs T3 vs T4: 60 vs 21% vs 17 vs 2% in the low-risk score group, 40 vs 29% vs 27 vs 4% in the high-risk score group.

Considering the complexity of the relationship between ferroptosis and HCC, we illustrated the workflow of risk score construction with the alluvial diagram (Figure 11A). These results indicated that cluster A was linked to a lower risk score, whereas B and C clusters exhibited higher risk scores (Figure 11A). We also explored the relationship between the risk score and HCC subtype and found that the A subtype was associated with the lowest risk score, and C subtype was associated with the highest risk score (Figure 11B). The relationship between the risk score and alive or dead status showed that the patients with dead status were associated with a higher risk score than those with alive status (Figure 11C). The relationship between the risk score and difference grades showed that the patients with a higher grade appeared to be associated with a higher risk score (Figure 11D). The relationship between the risk score and difference in tumor stages showed that the patients with higher difference in tumor stages appeared to be associated with a higher risk score (Figure 11E). The relationship between the risk score and difference TNM stages showed that the patients with higher difference in TNM stage appeared to be associated with a higher risk score (Figure 11F). The alive patients were confirmed with low-risk score than those with high-risk score (alive vs dead: 77 vs 23% in the low-risk score group and 56 vs 44% in the high-risk score group; Figure 11G). The lower grade was confirmed in patients with low-risk score compared to those with high-risk score (G1 vs G2 vs G3 vs G4: 16 vs 55% vs 27 vs 2% in the low-risk score group, 11 vs 43% vs 41 vs 5% in the high-risk score group; Figure 11H). A consistent result was also observed in the tumor stage (Figure 11I) and TNM stage (Figure 11J). Collectively, these results revealed that the risk score is related to the tumor stage and correlates with the prognosis of HCC patients.

### Building a Combined Nomogram for Clinical Practice

**FIGURE 12:**
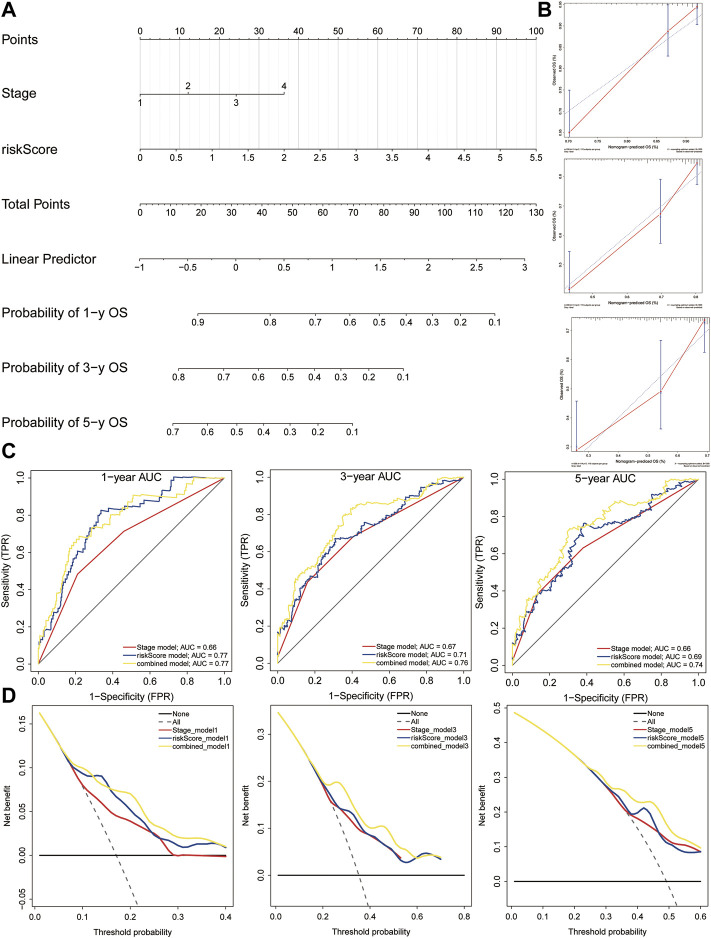
Nomogram construction. **(A)** Nomogram was constructed based on the risk score and clinical stage. **(B)** Calibration cures were plotted to assess the decision accuracy of the nomogram. **(C)** ROC curve analysis of the combined model, risk model, and stage model. **(D)** Decision curve analysis showing the relations between net benefit and threshold probability.

We built a comprehensive nomogram that could be applied in clinical practice based on the risk score and clinical stage (Figure 12A). Then, the calibration curves were performed to assess the prediction value of this nomogram (Figure 12B). The results demonstrated that the calibration curve for stage and risk score agreed well with the ideal observations. Furthermore, ROC analysis revealed that the combined nomogram showed the best performances in predicting the overall survival of HCC patients, compared to the risk score and clinical stage (AUC = 0.77 at 1 year, 0.76 at 3 years, and 0.74 at 5 years, Figure 12C). In addition, decision curve analysis indicated that the combined model achieved greater net benefit than the stage or risk score alone (Figure 12D).

## Discussion

The number of HCC patients and the mortality rates still show a growing trend globally (Couri and Pillai, 2019). Sorafenib resistance remains a big challenge in the short-term treatment and long-term prognosis of patients with HCC. Mounting evidence has shown that ferroptosis-related genes play an essential role in sorafenib resistance (Nie et al., 2018; Viveiros et al., 2019) and anticancer immunity (Angeli et al., 2019). Herein, this study analyzed 752 HCC patients and found a good indicator for prognosis prediction guidance, which is named a combined nomogram. The nomogram could predict the survival of patients with HCC.

In recent years, accumulating studies have reported several ferroptosis-related gene models in the pathogenesis of HCC. Kai Wen et al. obtained the ferroptosis-related genes from the FerrFb database and constructed a 10-gene signature via univariate and LASSO Cox regression (Wen et al., 2021). Hao Zhang et al. obtained the ferroptosis-related genes from the KEGG pathway and GSEA databases and built a 7-gene signature based on the univariate and LASSO Cox regression (Zhang et al., 2021). Jie-ying Liang et al. acquired 60 ferroptosis-related genes from the previous studies and constructed a 10-gene signature using the univariate and LASSO Cox regression (Liang et al., 2020). Lijun Xu et al. retrieved 173 ferroptosis-related genes and 120 pyroptosis-related genes from the FerrDb and GeneCards website and constructed a 13-gene signature through univariate and LASSO Cox regression (Xu et al., 2022). Jukun Wang et al. acquired 60 ferroptosis-related genes from the previous studies and constructed a 3-gene signature using the univariate and LASSO Cox regression (Wang J. et al., 2021). Tuo Deng et al. obtained 41 ferroptosis-related genes from the KEGG database and identified 8 genes associated with the methylation status and overall survival of HCC. Subsequently, the unsupervised consensus clustering was performed and established a 15-gene signature based on the differentially expressed genes between ferroptosis-H and ferroptosis-L groups via the univariate and LASSO Cox regression (Deng et al., 2021). There are many possibilities to extending the ferroptosis-related gene model. In the present study, 287 ferroptosis-related genes were retrieved form the FerrDb database and previous studies. Subsequently, a total of 58 ferroptosis-related DEGs were identified in TCGA cohort, and 25 DEGs were further confirmed to be significantly associated with the prognostic of HCC patients. Next, we conducted consensus clustering analyses of HCC samples from TCGA dataset based on the 25 ferroptosis-related genes. Subsequently, the overall and disease-free survival rates were examined in detail among three clusters using Kaplan–Meier curves in TCGA cohort. These results were further validated in the ICGC cohort. Taking immune infiltration into consideration will help us understand the role of ferroptosis-related genes. The A cluster was characterized by eosinophilia, the C cluster was characterized by effector memory CD4+/CD8+ T cells, activated CD4+/CD8+ T cells, activated B cells, activated dendritic cells, immature B cells, immature dendritic cells, MDSC, regulatory T cells, T follicular helper cells, and type 17 T helper cells. The three distinct HCC clusters were associated with the infiltration of immunoinflammatory cells and the expression of PD-L1 and TP53 mutation. Then, by the univariate Cox regression and random survival forest variable hunting (RSFVH) algorithm, the top 10 genes were ranked based on their predictive importance. Furthermore, because 10 genes could form a total of 2^10–1^ = 1,023 risk models, we performed Kaplan–Meier analysis to figure out the best risk model with relatively small genes and relatively significant *p* value. Hence, this is clearly different from other prediction models, including the LASSO risk model. Finally, a 5-gene signature was constructed and externally validated in several external datasets, including MED8, PIGU, PPM1G, RAN, and SNRPB. Furthermore, this result was validated by another independent cohort CPTAC in the protein level. According to the value of hazard ratio (HR), they were considered as the risk genes. Mediator complex subunit 8 (MED8) correlated with poorer overall survival and advanced clinical stage and showed higher expression in metastatic than primary tumors in clear cell RCC (Syring et al., 2016).Phosphatidylinositol glycan anchor biosynthesis class U (PIGU) may promote HCC progression by activating the NF-κB pathway (Wei et al., 2020) and could be an oncogene in HCC (Cao et al., 2019). PPM1G, protein phosphatase, Mg2+/Mn2+-dependent 1G, was reported to promote the progression of HCC by regulating the alternative splicing of SRSF3 (Chen et al., 2021). RAN, a member of RAS oncogene family, could promote the proliferation and migration ability of head and neck squamous cell carcinoma cells (Zhang et al., 2020). SNRPB, small nuclear ribonucleoprotein polypeptides B and B1, has been reported to promote HCC progression by inducing metabolic reprogramming (Chen et al., 2020). In general, these five genes in the signature were all cancer-promoting genes. While the effectiveness of a single clinical factor is insufficient, a multiple-factor signature may bring about changes in personalized HCC treatment. Consequently, a ferroptosis-related signature was constructed in the training group by univariate Cox regression, RSFVH algorithm, and Kaplan–Meier analyses. To make it suitable for the application in clinical HCC diagnosis, a nomogram was constructed to make better individualized treatment. This proposed model is one of the more realistic models than earlier models reported in the literature. We performed unsupervised cluster analysis based on 25 survival-related genes and examined in greater detail the differences in prognostic, immune cell infiltration, PD-L1 expression, and TP53 mutations among three subclusters. We collected more comprehensive resource of ferroptosis-related genes and constructed a more robust risk model using multiple machine learning methods.

Nevertheless, several limitations still remain in the present study. In this study, we only explored the correlation between ferroptosis-related genes and immune cell infiltration and PD-L1 and TP53 mutations and did not explain the role and underlying mechanisms of ferroptosis in the immune response of HCC. Therefore, further *in vivo* and *in vitro* experiments are necessary to validate these mechanisms.

In summary, our study demonstrated that a five-gene signature, and a combined nomogram can predict individual survival of patients with HCC. This study highlights the promising potential of the novel signature, the nomogram can function as the prognostic indicators for individual survival prediction and therapeutic decision-making.

## Data Availability

The original contributions presented in the study are included in the article/Supplementary Material, further inquiries can be directed to the corresponding authors.

## References

[B1] Angeli J. P. F. Krysko D. V. Conrad M. (2019). Ferroptosis at the Crossroads of Cancer-Acquired Drug Resistance and Immune Evasion. Nat. Rev. Cancer 19, 405–414. 10.1038/s41568-019-0149-1 31101865

[B2] Cao J. Wang P. Chen J. He X. (2019). PIGU Overexpression Adds Value to TNM Staging in the Prognostic Stratification of Patients with Hepatocellular Carcinoma. Hum. Pathol. 83, 90–99. 10.1016/j.humpath.2018.08.013 30171988

[B3] Capelletti M. M. Manceau H. Puy H. Peoc′h K. (2020). Ferroptosis in Liver Diseases: An Overview. Ijms 21, 4908. 10.3390/ijms21144908

[B4] Chen D. Zhao Z. Chen L. Li Q. Zou J. Liu S. (2021). PPM1G Promotes the Progression of Hepatocellular Carcinoma via Phosphorylation Regulation of Alternative Splicing Protein SRSF3. Cell Death Dis 12, 722. 10.1038/s41419-021-04013-y 34290239 PMC8295330

[B5] Chen W. Ma T. Zhang J. Zhang X. Chen W. Shen Y. (2020). A Systematic Review and Meta-Analysis of Adjuvant Transarterial Chemoembolization after Curative Resection for Patients with Hepatocellular Carcinoma. Hpb 22, 795–808. 10.1016/j.hpb.2019.12.013 31980307

[B6] Couri T. Pillai A. (2019). Goals and Targets for Personalized Therapy for HCC. Hepatol. Int. 13, 125–137. 10.1007/s12072-018-9919-1 30600478

[B7] Deng T. Hu B. Jin C. Tong Y. Zhao J. Shi Z. (2021). A Novel Ferroptosis Phenotype‐related Clinical‐molecular Prognostic Signature for Hepatocellular Carcinoma. J. Cel Mol Med 25, 6618–6633. 10.1111/jcmm.16666

[B8] Gorgen A. Galvin Z. Huang A. C. Vinaixa C. O′Rourke J. M. Francoz C. (2020). The Impact of Direct-Acting Antivirals on Overall Mortality and Tumoral Recurrence in Patients with Hepatocellular Carcinoma Listed for Liver Transplantation: An International Multicenter Study. Transplantation 104, 2087–2096. 10.1097/TP.0000000000003115 31978002

[B9] Hänzelmann S. Castelo R. Guinney J. (2013). GSVA: Gene Set Variation Analysis for Microarray and RNA-Seq Data. BMC Bioinformatics 14, 7. 10.1186/1471-2105-14-7 23323831 PMC3618321

[B10] Hilmi M. Neuzillet C. Calderaro J. Lafdil F. Pawlotsky J.-M. Rousseau B. (2019). Angiogenesis and Immune Checkpoint Inhibitors as Therapies for Hepatocellular Carcinoma: Current Knowledge and Future Research Directions. J. Immunotherapy Cancer 7, 333. 10.1186/s40425-019-0824-5

[B11] Kudo M. Finn R. S. Qin S. Han K.-H. Ikeda K. Piscaglia F. (2018). Lenvatinib versus Sorafenib in First-Line Treatment of Patients with Unresectable Hepatocellular Carcinoma: a Randomised Phase 3 Non-inferiority Trial. The Lancet 391, 1163–1173. 10.1016/S0140-6736(18)30207-1

[B12] Kulik L. El-Serag H. B. (2019). Epidemiology and Management of Hepatocellular Carcinoma. Gastroenterology 156, 477–491. 10.1053/j.gastro.2018.08.065 30367835 PMC6340716

[B13] Li J. Cao F. Yin H.-l. Huang Z.-j. Lin Z.-t. Mao N. (2020). Ferroptosis: Past, Present and Future. Cel Death Dis 11, 88. 10.1038/s41419-020-2298-2

[B14] Liang J.-y. Wang D.-s. Lin H.-c. Chen X.-x. Yang H. Zheng Y. (2020). A Novel Ferroptosis-Related Gene Signature for Overall Survival Prediction in Patients with Hepatocellular Carcinoma. Int. J. Biol. Sci. 16, 2430–2441. 10.7150/ijbs.45050 32760210 PMC7378635

[B15] Liu Z. Guo C. Dang Q. Wang L. Liu L. Weng S. (2022a). Integrative Analysis from Multi-center Studies Identities a Consensus Machine Learning-Derived lncRNA Signature for Stage II/III Colorectal Cancer. EBioMedicine 75, 103750. 10.1016/j.ebiom.2021.103750 34922323 PMC8686027

[B16] Liu Z. Guo C. Li J. Xu H. Lu T. Wang L. (2021a). Somatic Mutations in Homologous Recombination Pathway Predict Favourable Prognosis after Immunotherapy across Multiple Cancer Types. Clin. Translational Med 11, e619. 10.1002/ctm2.619

[B17] Liu Z. Liu L. Guo C. Yu S. Meng L. Zhou X. (2021b). Tumor Suppressor Gene Mutations Correlate with Prognosis and Immunotherapy Benefit in Hepatocellular Carcinoma. Int. Immunopharmacology 101, 108340. 10.1016/j.intimp.2021.108340

[B18] Liu Z. Liu L. Weng S. Guo C. Dang Q. Xu H. (2022b). Machine Learning-Based Integration Develops an Immune-Derived lncRNA Signature for Improving Outcomes in Colorectal Cancer. Nat. Commun. 13, 816. 10.1038/s41467-022-28421-6 35145098 PMC8831564

[B19] Liu Z. Wang Q. Wang X. Xu Z. Wei X. Li J. (2020). Circular RNA cIARS Regulates Ferroptosis in HCC Cells through Interacting with RNA Binding Protein ALKBH5. Cell Death Discov. 6, 72. 10.1038/s41420-020-00306-x 32802409 PMC7414223

[B20] Liu Z. Xu H. Weng S. Ren Y. Han X. (2022c). Stemness Refines the Classification of Colorectal Cancer with Stratified Prognosis, Multi-Omics Landscape, Potential Mechanisms, and Treatment Options. Front. Immunol. 13, 828330. 10.3389/fimmu.2022.828330 35154148 PMC8828967

[B21] Llovet J. M. Ricci S. Mazzaferro V. Hilgard P. Gane E. Blanc J.-F. (2008). Sorafenib in Advanced Hepatocellular Carcinoma. N. Engl. J. Med. 359, 378–390. 10.1056/NEJMoa0708857 18650514

[B22] Louandre C. Ezzoukhry Z. Godin C. Barbare J.-C. Mazière J.-C. Chauffert B. (2013). Iron-dependent Cell Death of Hepatocellular Carcinoma Cells Exposed to Sorafenib. Int. J. Cancer 133, 1732–1742. 10.1002/ijc.28159 23505071

[B23] Nie J. Lin B. Zhou M. Wu L. Zheng T. (2018). Role of Ferroptosis in Hepatocellular Carcinoma. J. Cancer Res. Clin. Oncol. 144, 2329–2337. 10.1007/s00432-018-2740-3 30167889 PMC11813439

[B24] Ritchie M. E. Phipson B. Wu D. Hu Y. Law C. W. Shi W. (2015). Limma powers Differential Expression Analyses for RNA-Sequencing and Microarray Studies. Nucleic Acids Res. 43, e47. 10.1093/nar/gkv007 25605792 PMC4402510

[B25] Subramanian A. Tamayo P. Mootha V. K. Mukherjee S. Ebert B. L. Gillette M. A. (2005). Gene Set Enrichment Analysis: a Knowledge-Based Approach for Interpreting Genome-wide Expression Profiles. Proc. Natl. Acad. Sci. U.S.A. 102, 15545–15550. 10.1073/pnas.0506580102 16199517 PMC1239896

[B26] Sun X. Ou Z. Chen R. Niu X. Chen D. Kang R. (2016). Activation of the P62-Keap1-NRF2 Pathway Protects against Ferroptosis in Hepatocellular Carcinoma Cells. Hepatology 63, 173–184. 10.1002/hep.28251 26403645 PMC4688087

[B27] Syring I. Klümper N. Offermann A. Braun M. Deng M. Boehm D. (2016). Comprehensive Analysis of the Transcriptional Profile of the Mediator Complex across Human Cancer Types. Oncotarget 7, 23043–23055. 10.18632/oncotarget.8469 27050271 PMC5029609

[B28] Tabrizian P. Jibara G. Shrager B. Schwartz M. Roayaie S. (2015). Recurrence of Hepatocellular Cancer after Resection. Ann. Surg. 261, 947–955. 10.1097/SLA.0000000000000710 25010665

[B29] Viveiros P. Riaz A. Lewandowski R. J. Mahalingam D. (2019). Current State of Liver-Directed Therapies and Combinatory Approaches with Systemic Therapy in Hepatocellular Carcinoma (HCC). Cancers 11, 1085. 10.3390/cancers11081085

[B30] Wang J. Han K. Zhang C. Chen X. Li Y. Zhu L. (2021). Identification and Validation of Ferroptosis-Associated Gene-Based on Immune Score as Prognosis Markers for Hepatocellular Carcinoma Patients. J. Gastrointest. Oncol. 12, 2345–2360. 10.21037/jgo-21-237 34790397 PMC8576212

[B31] Wang Q. Bin C. Xue Q. Gao Q. Huang A. Wang K. (2021). GSTZ1 Sensitizes Hepatocellular Carcinoma Cells to Sorafenib-Induced Ferroptosis via Inhibition of NRF2/GPX4 axis. Cel Death Dis 12, 426. 10.1038/s41419-021-03718-4

[B32] Wei X. Yang W. Zhang F. Cheng F. Rao J. Lu L. (2020). PIGU Promotes Hepatocellular Carcinoma Progression through Activating NF-Κb Pathway and Increasing Immune Escape. Life Sci. 260, 118476. 10.1016/j.lfs.2020.118476 32971102

[B33] Wen K. Yan Y. Shi J. Hu L. Wang W. Liao H. (2021). Construction and Validation of a Combined Ferroptosis and Hypoxia Prognostic Signature for Hepatocellular Carcinoma. Front. Mol. Biosci. 8, 809672. 10.3389/fmolb.2021.809672 34977159 PMC8719198

[B34] Wilkerson M. D. Hayes D. N. (2010). ConsensusClusterPlus: a Class Discovery Tool with Confidence Assessments and Item Tracking. Bioinformatics 26, 1572–1573. 10.1093/bioinformatics/btq170 20427518 PMC2881355

[B35] Xu L. Zheng Q. Liu W. (2022). Combination of Ferroptosis and Pyroptosis to Construct a Prognostic Classifier and Predict Immune Landscape, Chemotherapeutic Efficacy and Immunosuppressive Molecules in Hepatocellular Carcinoma. BMC Cancer 22, 229. 10.1186/s12885-022-09301-0 35236323 PMC8892773

[B36] Yu G. Wang L.-G. Han Y. He Q.-Y. (2012). clusterProfiler: an R Package for Comparing Biological Themes Among Gene Clusters. OMICS: A J. Integr. Biol. 16, 284–287. 10.1089/omi.2011.0118

[B37] Zhang C. Zhao X. Du W. Shen J. Li S. Li Z. (2020). Ran Promotes the Proliferation and Migration Ability of Head and Neck Squamous Cell Carcinoma Cells. Pathol. - Res. Pract. 216, 152951. 10.1016/j.prp.2020.152951 32334891

[B38] Zhang H. Liu R. Sun L. Guo W. Hu X. (2021). The Effect of Ferroptosis-Related Genes on Prognosis and Tumor Mutational Burden in Hepatocellular Carcinoma. J. Oncol. 2021, 1–12. 10.1155/2021/7391560

