# Supplementary material for: The Prognostic Significance and Potential Mechanism of Ferroptosis-Related Genes in Hepatocellular Carcinoma
Source: Front Genet. 2022 Apr 26;13:844624. doi: 10.3389/fgene.2022.844624 (PMC9086291; doi:10.3389/fgene.2022.844624)
Supplement: Supplementary file 1 [file Presentation1.PDF]

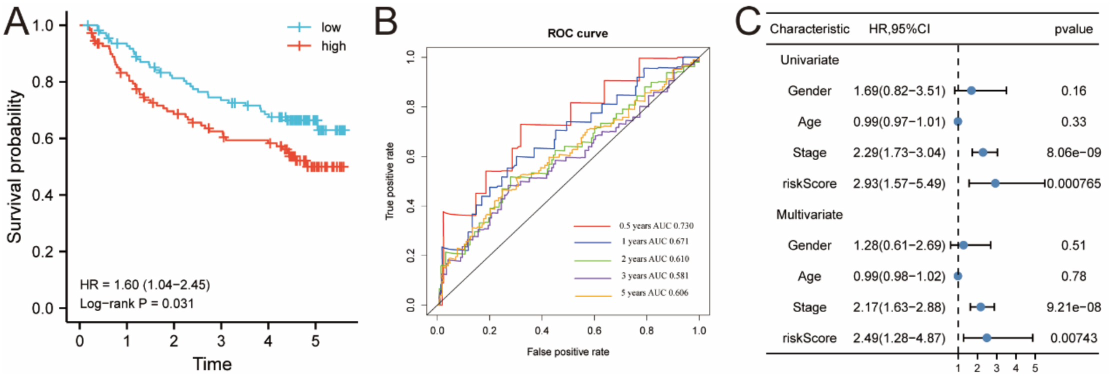

Figure S1. The predictive power of the risk model in GSE14520 cohort. A. Survival curves were plotted using the Kaplan–Meier method. B. Receiver operating characteristic curve was applied to assess the performance of the risk model. C. Univariate and multivariate Cox regression analysis were performed.
